# Temporal changes in the positivity rate of common enteric viruses among paediatric admissions in coastal Kenya, during the COVID-19 pandemic, 2019–2022

**DOI:** 10.1186/s13099-023-00595-4

**Published:** 2024-01-04

**Authors:** Arnold W. Lambisia, Nickson Murunga, Martin Mutunga, Robinson Cheruiyot, Grace Maina, Timothy O. Makori, D. James Nokes, Charles N. Agoti

**Affiliations:** 1 Kenya Medical Research Institute (KEMRI) - Wellcome Trust Research Programme (KWTRP), P.O. Box 230, Kilifi, 80108 Kenya; 2 School of Life Sciences and Zeeman Institute for Systems Biology and Infectious Disease Epidemiology Research (SBIDER), University of Warwick, Coventry, CV4 7AL UK; 3 Department of Public Health, Pwani University, P.O. Box 195, Kilifi, 80108 Kenya

**Keywords:** COVID-19, Rotavirus group A, Norovirus GII, Astrovirus, Sapovirus, Adenovirus F40/41, Positivity rate, Kenya, Paediatrics

## Abstract

**Background:**

The non-pharmaceutical interventions (NPIs) implemented to curb the spread of severe acute respiratory syndrome coronavirus 2 (SARS-CoV-2) early in the coronavirus disease 2019 (COVID-19) pandemic, substantially disrupted the activity of other respiratory viruses. However, there is limited data from low-and-middle income countries (LMICs) to determine whether these NPIs also impacted the transmission of common enteric viruses. Here, we investigated the changes in the positivity rate of five enteric viruses among hospitalised children who presented with diarrhoea to a referral hospital in coastal Kenya, during COVID-19 pandemic period.

**Methods:**

A total of 870 stool samples from children under 13 years of age admitted to Kilifi County Hospital between January 2019, and December 2022 were screened for rotavirus group A (RVA), norovirus genogroup II (GII), astrovirus, sapovirus, and adenovirus type F40/41 using real-time reverse-transcription polymerase chain reaction. The proportions positive across the four years were compared using the chi-squared test statistic.

**Results:**

One or more of the five virus targets were detected in 282 (32.4%) cases. A reduction in the positivity rate of RVA cases was observed from 2019 (12.1%, 95% confidence interval (CI) 8.7–16.2%) to 2020 (1.7%, 95% CI 0.2–6.0%; p *< 0.001*). However, in the 2022, RVA positivity rate rebounded to 23.5% (95% CI 18.2%–29.4%). For norovirus GII, the positivity rate fluctuated over the four years with its highest positivity rate observed in 2020 (16.2%; 95% C.I, 10.0–24.1%). No astrovirus cases were detected in 2020 and 2021, but the positivity rate in 2022 was similar to that in 2019 (3.1% (95% CI 1.5%–5.7%) vs. 3.3% (95% CI 1.4–6.5%)). A higher case fatality rate was observed in 2021 (9.0%) compared to the 2019 (3.2%), 2020 (6.8%) and 2022 (2.1%) (*p* < 0.001).

**Conclusion:**

Our study finds that in 2020 the transmission of common enteric viruses, especially RVA and astrovirus, in Kilifi Kenya may have been disrupted due to the COVID-19 NPIs. After 2020, local enteric virus transmission patterns appeared to return to pre-pandemic levels coinciding with the removal of most of the government COVID-19 NPIs.

**Supplementary Information:**

The online version contains supplementary material available at 10.1186/s13099-023-00595-4.

## Introduction

Although water sanitation and hygiene (WASH) programmes and new vaccine introductions have resulted in significant reductions of paediatric diarrhoea morbidity and mortality globally, virus-associated diarrhoea is still a major cause of hospital admissions in several low and middle-income settings [1]. In 2019, approximately 300,000 deaths were recorded globally in children below 14 years of age due to rotavirus group A (RVA), norovirus GI and GII, and adenovirus F40/41 infections [2].

Following the emergence of severe acute respiratory syndrome coronavirus 2 (SARS-CoV-2), the aetiological agent of coronavirus disease 2019 (COVID-19), several reports have indicated perturbations in the epidemiology of common enteric viruses associated with diarrhoeal disease. For instance, in France [3], Poland [4], China [5, 6] and USA [7], the prevalence of RVA during the year 2020 was lower compared to 2018 and 2019. However, in 2021 there was a surge of RVA cases in these countries. Like RVA, a decrease in cases of norovirus GII, sapovirus, adenovirus F40/41 and astrovirus was reported in 2020 in Spain [8] and Korea [9] compared to previous years. However, this decrease in virus detection has been followed by remarkable outbreaks in 2021 [5, 10]. Sporadic outbreaks of norovirus have also been reported in China in September 2020 and in the USA, where a total of 992 norovirus outbreaks were reported between August 2021 and July 2022 [5, 11, 12].

The decline of the detection rates of some of the enteric viruses in the early COVID-19 pandemic phase has been postulated to be a result of the stringent non-pharmaceutical interventions (NPIs) that were implemented to abrogate the pandemic [13]. Some of the measures included those that may impact enteric pathogen transmission such as frequent hand washing, increased hygiene, social distancing, closure of restaurants and restricted movement either locally or internationally [13].

In coastal Kenya, the prevalence of enteric viruses over the past decade has been closely monitored through a hospital-based surveillance [14]. No significant change in the prevalence has been detected for all enteric viruses except sapovirus (7.6% vs. 4.0%, *p value* < 0.05) pre-post rotavirus vaccine introduction in July 2014 [14]. RVA positivity in hospital admissions decreased significantly only among ELISA detected cases but not RT-PCR detected cases [15]. Continuous monitoring of these enteric viruses is key in providing insights on their epidemiology for disease management and informing public health policy. In this study, we aimed to describe the epidemiological patterns of 5 common enteric viruses associated with diarrhoea during the period spanning the COVID-19 pandemic.

## Methods

### Study site and population

This study was undertaken as part of our routine surveillance of RVA infections in Kilifi County Hospital (KCH), Kenya [14, 16, 17]. To be recruited, a participant had to satisfy the following criteria: (a) admitted with diarrhoea (passing of ≥ three loose stools within the last 24 h) as one of their illness symptom(s) [18], (b) aged < 13-year-old, (c) consent given from a parent or guardian to be in the study [14, 16, 17]. The surveillance started in 2009 and has continued to date (2023). In this analysis we focused on participants recruited between 1st January 2019 and 31st December 2022.

### Laboratory methods

#### Molecular testing for common enteric viruses

##### Total nucleic acid (TNA) extraction

TNA was extracted from 0.2 g of stool (or 200ul if liquid) using the QIAamp Fast DNA Stool Mini kit (Qiagen, Manchester, UK) and eluted in 200ul of elution buffer as previously described [14, 16].

##### Virus (RT)-PCR screening

The extracted TNA was combined with the TaqMan Fast Virus 1-step master mix and virus specific primers (Additional file 1: Table S1) for each of the five viruses [16, 19] and processed on a real-time Quantistudio 5-flex instrument. The reaction mix comprised 2.5 µl of the TaqMan master mix, 1.2 µl of the primer-probe mix, 3.8 µl of nuclease free water and 5 µl of TNA. The thermocycling conditions were as follows; 95 °C for 20 s and 35 cycles of 94 °C for 15 s and 60 °C for 30 s. A cycle threshold cut-off of < 35.0 was applied to define virus positive samples for all targets screened.

### RVA genotyping

TNA from RVA positives were amplified using VP4 and VP7 segment specific primers, sequenced on the Illumina Miseq platform as previously described [20]. VP4 and VP7 segments were assembled from the short read data using a *de novo* assembly approach as previously described [20]. RVA genotypes were assigned using either BLAST or an online RVA genotyping tool [21]. The accession numbers for the RVA genotype data are MZ096489 to MZ096854.

### Statistical analysis

All statistical analysis was undertaken using R version 4.1.1 (2021-08-10). The level of government intervention was summarised using the Oxford Stringency index (SI), a composite measure based on nine response indicators including school closures, workplace closures, and travel bans, rescaled to a value from 0 to 100 (100 = strictest) [22]. Local stringency measures have been highlighted elsewhere [23] and summarized in Additional file 1: Table S2.

The virus positivity rate during each year was calculated as the proportion of samples that tested positive for the given virus given the total number of samples tested in the defined year. The data from 2019 has been previously reported elsewhere and formed a reference base of the situation before COVID-19 [16]. Comparisons across different years and groups were done using the chi-squared test statistic. Kruskal Wallis and Wilcoxon rank-sum tests were used to compare the distribution of continuous variables. Disease severity was estimated using the Vesikari Clinical Severity Scoring System Manual as previously described [16, 24].

## Results

### Basic demographic characteristics

Between January 2019 and December 2022, 1,613 patients aged under 13 years presented with diarrhoea as one of their illness symptoms at KCH. Of these, 870 (54.0%) consented enrolment into the study, gave a stool sample, and were included in this analysis. The reasons for missed sample collection in the study were: consent refusal (n = 344), other (n = 133), death (n = 68), discharged before sample collection(n = 35) and transferred before sample collection (n = 3).

All the 870 stool samples were screened for the five common enteric viruses. The majority of the recruited patients were in their first year of life (n = 371, 42.6%) and all suffered moderate-to-severe diarrhoeal disease (Table 1). The characteristics of the observed cases across the four years in terms of gender and age were similar (*p value > 0.05*). However, fatal outcome appeared more likely to occur in 2021 (9.0%) compared to 2019 (3.2%), 2020 (6.8%) and 2022 (2.1%) (*p value* < 0.001, Table 1). Less severe disease was also reported in 2020 compared to the other three years, Table 1.

**Table 1:** Demographic characteristics of children under 13 years admitted to Kilifi County Hospital, Kenya between January 2019 and December 2022

	2019 (n = 314) *	2020 (n = 117)	2021 (n = 201)	2022 (n = 238)	Total (n = 870)	P value
**Sex**						0.586
Female	137 (43.6%)	45 (38.5%)	81 (40.3%)	107 (45.0%)	370 (42.5%)	
Male	177 (56.4%)	72 (61.5%)	120 (59.7%)	131 (55.0%)	500 (57.5%)	
**Median age in months (Interquartile range)**	14.0 (8.0–25.0)	13.7 (8.1–27.0)	13.0 (7.5–23.4)	13.2 (8.5–21.3)	13.7 (8.1–23.0)	
**Age strata (months)**						0.252
<12	128 (40.8%)	51 (43.6%)	89 (44.3%)	103 (43.3%)	371 (42.6%)	
12–23	102 (32.5%)	36 (30.8%)	64 (31.8%)	86 (36.1%)	288 (33.1%)	
24–59	56 (17.8%)	13 (11.1%)	33 (16.4%)	35 (14.7%)	137 (15.7%)	
>60	28 (8.9%)	17 (14.5%)	15 (7.5%)	14 (5.9%)	74 (8.5%)	
**Outcome**						< 0.001
Alive	276 (87.9%)	109 (93.2%)	183 (91.0%)	231 (97.1%)	799 (91.8%)	
Dead	10 (3.2%)	8 (6.8%)	18 (9.0%)	5 (2.1%)	41 (4.7%)	
Data missing	28 (8.9%)	0 (0.0%)	0 (0.0%)	2 (0.8%)	30 (3.4%)	
**Disease severity**						0.01
Moderate	90 (28.7%)	47 (40.2%)	45 (22.4%)	65 (27.3%)	247 (28.4%)	
Severe	224 (71.3%)	70 (59.8%)	156 (77.6%)	173 (72.7%)	623 (71.6%)	

*Pre-pandemic data has been reported in an earlier manuscript [16]

### Trends in diarrhoeal cases in the context of the COVID-19 pandemic

**Fig. 1:**
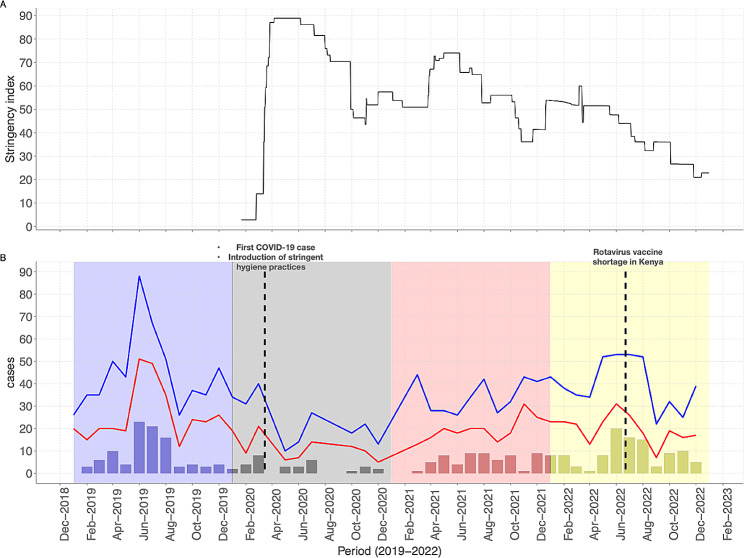
(**A**) COVID-19 stringency index quantifying the government NPI measures aimed to curb the spread of SARS-CoV-2. (*Source*https://ourworldindata.org/covid-stringency-index). (**B**) Temporal trends of monthly diarrhoea and virus cases between January 2019 and December 2022. The blue and red line graphs show the total eligible and recruited diarrhoea cases respectively, while the bar graphs show the total virus positive cases per month

After the initial detection of the first COVID-19 case in Kenya on 12th March 2020 [25], the government implemented a range of NPIs to curb the pandemic (summarised in Fig. 1a using the Oxford Stringency index and local restrictions listed in supplementary Table 2). The trend of monthly recorded diarrhoea cases recruited into our surveillance between January 2019 and December 2022 is shown in Fig. 1b. The highest monthly peak in cases was recorded in 2019 before the COVID-19 pandemic. The lowest number of diarrhoea cases was in the 2020, but a gradual rebound was observed in 2021 and 2022.

### Single virus Infections and coinfections

At least one of the five virus targets were detected in 282 (32.4%) cases. The proportion of samples positive for the five viruses we tested in the stool samples for the different years is summarised in Table 1. The positivity rate of RVA deeped in 2020, at 1.7% (95% C.I, 0.2–6.0%) compared to 2019 (12.1% (95% C.I, 8.7–14.9%) and gradually rose in 2021 (16.9% (95% C.I, 12.0 − 22.8%)) and 2022 (23.5% (95% C.I, 18.2 − 29.4%)) and the differences were statistically significant (p value < 0.001) (Table 2).

The norovirus GII positivity rate fluctuated over the four years and the difference was statistically significant (*p = 0.04*) (Table 2). The highest positivity rate for norovirus GII was 16.2% (95% C.I, 10.0–24.1%) in 2020 and lowest at 6.4% (95% C.I, 3.4–10.8%) in 2021 (Table 2). No astrovirus cases were detected in the 2020 and 2021, but the cases in 2022 (3.3%) with a similar positivity rate to what was reported in 2019 (3.1%, Table 2). The positivity rates for sapovirus and adenovirus type F40/41 did not change across the three phases (*p value* > 0.05, χ2).

**Table 2:** Comparison of the detection rates of five common enteric virus between January 2019 and December 2022

	Total (n = 870)	2019 (n = 314)		2020 (n = 117)		2021 (n = 201)		2022 (n = 238)		P value
		Cases	Proportion (95% CI)	Cases	Proportion (95% CI)	Cases	Proportion (95% CI)	Cases	Proportion (95% CI)	
**Virus**										
Rotavirus	130 (14.9%)	38	12.1 (8.7–16.2)	2	1.7 (0.2–6.0)	34	16.9 (12.0–22.8)	56	23.5 (18.2–29.4)	< 0.001
Norovirus GII	89 (10.2%)	34	10.8 (7.6–14.8)	19	16.2 (10.0–24.1)	13	6.4 (3.4–10.8)	23	9.6 (6.2–14.1)	0.04
Astrovirus	18 (2.1%)	10	3.1 (1.5–5.7)	0	–	0	–	8	3.3 (1.4–6.5)	0.01
Sapovirus	33 (3.8%)	11	3.5 (1.7–6.1)	3	2.5 (0.5–7.3)	7	3.4 (1.4–7.0)	12	5.0 (2.6–8.6)	0.65
Adenovirus F40/41	30 (3.4%)	9	2.8 (1.3– 5.3)	8	6.8 (2.9–13.0)	6	2.9 (1.1–6.3)	7	2.9 (1.1–5.9)	0.19

Between 2019 and 2022, 13 samples had a coinfection of two or more viruses of the screened viruses. The most common coinfections were RVA and norovirus GII (n = 4), adenovirus 40/41 and astrovirus (n = 3), and adenovirus 40/41 and sapovirus (n = 3) (Additional file 1: Table S3).

### Monthly virus trends

**Fig. 2:**
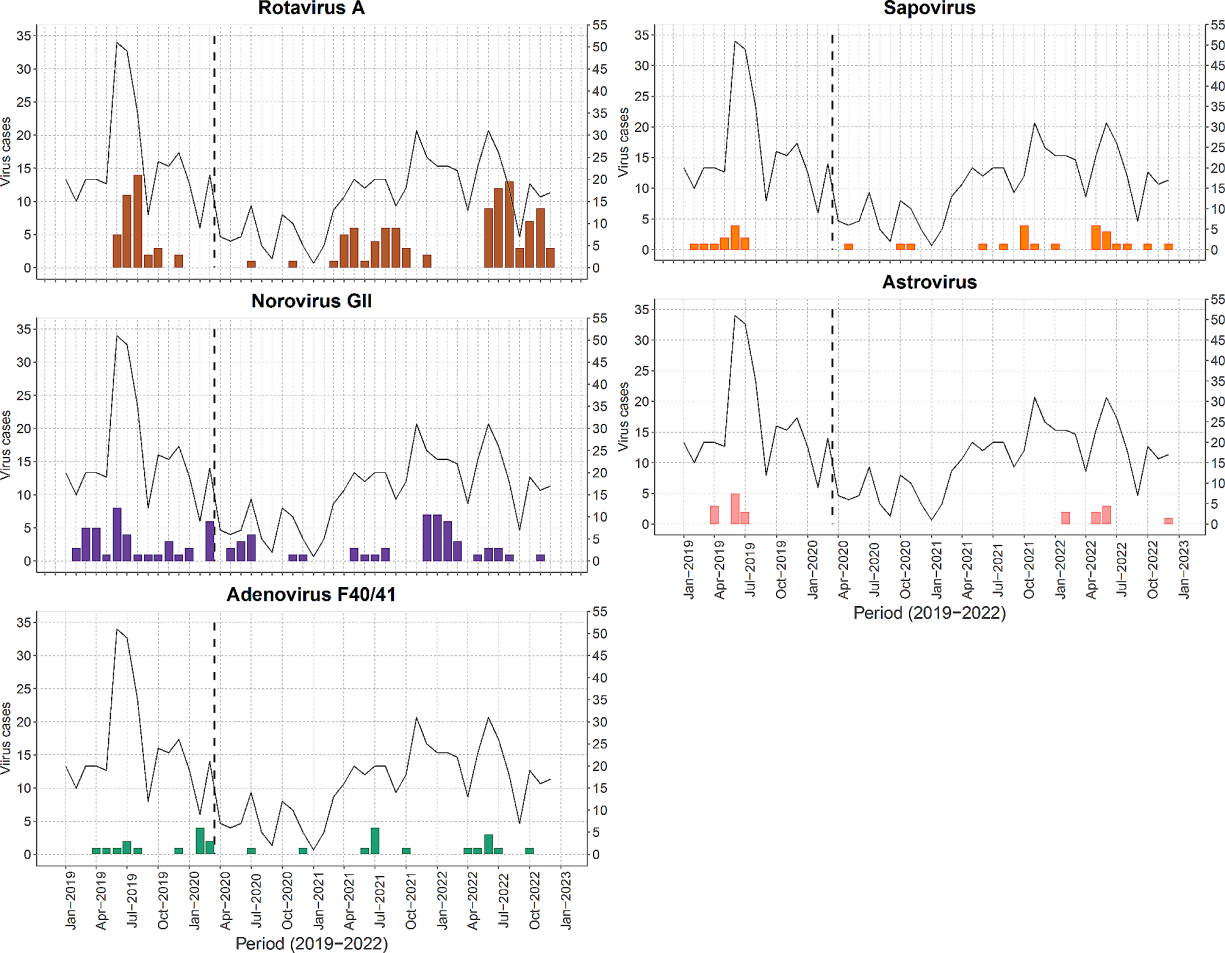
Monthly temporal distribution of common enteric virus cases in children under 13 years admitted to KCH with diarrhoea between January 2019 and December 2022. The black trendline shows the monthly diarrhoeal cases over time and the vertical dotted line represents when the first COVID-19 case was detected in Kenya

In all the years, peak RVA cases were observed in the month of August, except in 2020 where only two RVA cases were detected, Fig. 2. The peak months for norovirus GII varied across the different years. Less than five cases were reported over the four years for adenovirus F40/41, sapovirus and astrovirus in each month. In 2020 and 2021, no astrovirus cases were detected but it re-emerged in 2022 (Fig. 2).

### RVA genotypes and vaccination status of positive cases

Of the 130 RVA positive detected during the study, 87 (66.9%) and 70 (53.8%) successively sequenced in the VP7 and VP4 segment, respectively. The years 2019, 2020 and 2021 were predominated by the G3P[8] genotype (n = 48, 80.0%). In 2022, we observed replacement of the G3P[8] genotype with multiple genotypes: G2P[4] (n = 2), G9P[8] (n = 10), and G9P[4] (n = 4). To note, in the 2022 there was some incomplete genotyping due to failed sequencing in the VP4 and VP7 segments G2P[x] (n = 5) and GxP[8] (n = 8).

In 2019, 2020, 2021 and 2022, 73.8%, 67.5%, 65.1% and 66.8% of the participants had received at least one dose of the Rotarix® vaccine. Among the 130 RVA positive cases, 88 (67.7%) had received at least one dose of the Rotarix® vaccine two doses, 18 (12.9%) had not received a vaccine and 24 (16.9%) had no vaccination records. One participant was RVA positive five days after receiving a Rotarix dose and their sample genotyped as G3P[8].

### Disease outcome

In the study, a total of 41 (4.7%) cases succumbed among the 870 that we analysed. Only nine of these cases were positive for at least of the viruses we tested i.e., norovirus GII (n = 4), adenovirus type F40/41 (n = 2), RVA (n = 1), sapovirus (n = 1) and a coinfection (RVA & sapovirus, n = 1) (Additional file 1: Table S3). The other 32 cases had none of the five viruses detected.

## Discussion

Our study observed a decrease in paediatric diarrhoea admissions to a coastal Kenya county referral hospital in the first year of the COVID-19 pandemic (2020) compared to the pre-pandemic year (2019). This may have occurred due to the government NPIs such as (a) restriction of people movement limiting healthcare usage, (b) increased hygiene practices that may have limited transmission of the enteric pathogens [8] among other reasons. Further there was a health worker strike in December 2020.

Previous data shows that in this region, diarrhoea cases usually peak in June–July of every year [16]. Both in the years 2020 and 2021, the months of June and July coincided with high stringency measures in the country [23]. Importantly, after March 2021, targeted COVID-19 vaccination campaigns started replacing NPIs and by August 2021, the government had dropped most of the NPI measures such as closure of schools, curfews, lockdowns and restriction of public gatherings and restrictions in public transport.

While effective rotavirus vaccines are available, RVA remains the number one cause of paediatric diarrhoea admissions in many LMICs [26]. Notably, our study report a significant decrease in RVA positivity rate in 2020 compared to 2019 followed by a rebound in 2021 and 2022, a finding consistent with other studies elsewhere [4, 6–8]. Notably, the positivity rate of RVA in 2022 was higher compared to all the previous years. Such a resurgence of RVA activity was also been observed elsewhere e.g., in Hong Kong after the first year of the pandemic [5].

The return and apparent increase of RVA activity in the 2021 and 2022 can be attributed in a number of factors e.g., the gradual relaxation of the COVID-19 NPIs, the reduction of RVA population immunity during the first year of the pandemic due to the limited circulation of the virus, circulation of RVA strains heterologous to the Rotarix® vaccine in 2021 and 2022 (e.g. G9P[8], G9P[4] and G2P[4]). Some of these strains have been noted to have limited cross-reactivity with the Rotarix G1P[8] strain [27]. Further, vaccination delays and Rotarix® vaccine stockouts that occurred in between June 2022 and January 2023 when a vaccine switch to Rotavac® was made in Kenya [28]. The introduction of G9 genotypes in Malaysia was associated with an increase RVA prevalence [29] similar to what we observed in coastal Kenya 2022 whereby the G3P[8] was replaced by the G9P[8], G9P[4] and G2P[4] genotypes.

Our previous analysis indicated that in Kilifi, the prevalence of norovirus GII increased post-rotavirus vaccine introduction [14, 16]. Strikingly, the positivity rate of norovirus GII was highest in 2020 phase compared to other years. It appeared that norovirus activity was unimpacted by the NPI measures. Norovirus is known to be highly contagious infectious agent that requires an exceptionally small number of viral particles to transmit to susceptible individuals [30]. We hypothesize that the persistent norovirus GII activity even in the face of the pandemic NPIs may is indicative local transmission of a new strain that that the population was less immune to and reflected the resistance of norovirus inactivation to common disinfecting agents such as hypochlorite and 70% ethanol that were commonly used during the pandemic [31]. An increase in norovirus activity and multiple sporadic outbreaks have reported in the USA, China and South Africa during the COVID-19 period [5, 11, 12, 32].

While astrovirus, sapovirus, and adenovirus F40/41 are among the most common causes of diarrhoea, their prevalence is much lower than that of RVA and norovirus [14, 16]. Astrovirus was not detected in the 2020 and 2021 but re-emerged in the 2022 with a positivity rate similar to what was observed in 2019. Astrovirus and sapovirus detection on the Kenyan coast has been always characterised by very low prevalence (< 5%) [14, 16]. The non-detection of astrovirus in 2020 and 2021 does not necessarily imply it was out of circulation but if its transmission decreased, it will have required a larger sample size that that utilised in this study to detect it. This we cannot confirm that the virus was completely out of circulation during 2020 and 2021 [14, 16].

In China, enteric virus coinfections especially with RVA and norovirus was associated with severe diarrhoeal disease [33]. Similarly in coastal Kenya, a coinfection of RVA and norovirus GII coincided with severe disease. However, its key to note that 72% of the participants in the study presented with severe diarrhoeal disease. In 2020, there was a significant difference in mortality rate (9.0%) compared to the 2019 (3.2%), 2021 (6.8%) and 2022 (2.1). We hypothesize that the high mortality rate in the 2020 and partly 2021 may have been driven by the challenges with timely access to the hospitals and health care services during the pandemic, background circulation and potential infection with SARS-CoV-2 of for some of the patients seeking care for diarrhoeal disease [34].

This study had several limitations. We did not analyse healthy controls from the same population to adjust the aetiological fraction for asymptomatic carriage in the population. With the small number of detections for some of the virus targets, it hard to confidently infer seasonality without a larger study. There was substantial data missingness e.g., on deaths and almost half of the eligible participants refused consent to be in the study. For a conclusive inference of changes of the incidence of these viruses before during and after the pandemic, a population-based study with a clear population-based denominator is necessary.

In conclusion, our study observed a decrease in total diarrhoea admissions and enteric virus activity during in 2020 and 2021. However, in 2022 an increase in RVA and astrovirus activity is restored to pre-pandemic positivity rate or even higher. Our continuous enteric virus surveillance contributes understanding to the temporal changes in positivity rate of these viruses following the COVID-19 pandemic and provides important data to inform future public health policy.

### Electronic supplementary material

Below is the link to the electronic supplementary material.

**Supplementary Material 1:** Primer and probe sequences used in the detection of five common enteric viruses

## Data Availability

The datasets used and/or analyzed during the current study are available from the KWTRP Research repository via 10.7910/DVN/EG6MEH.
