# Supplementary material for: Temporal changes in the positivity rate of common enteric viruses among paediatric admissions in coastal Kenya, during the COVID-19 pandemic, 2019–2022
Source: Gut Pathog. 2024 Jan 4;16:2. doi: 10.1186/s13099-023-00595-4 (PMC10765698; doi:10.1186/s13099-023-00595-4)
Supplement: Supplementary file 1 — Supplementary Material 1: Primer and probe sequences used in the detection of five common enteric viruses [file 13099_2023_595_MOESM1_ESM.docx]

**Table S1: Primer and probe sequences**

| **Virus** | **Strand** | **Sequence** |
| --- | --- | --- |
| **Sapovirus** | Primer-F | CAGGCTCTCGCCACCTAC |
|  | Primer-R | CCCTCCATYTCAAACACTAWTTT |
|  | Probe | TGGTTCATAGGTGGTRC |
| **Astrovirus** | Primer-F | TCAACGTGTCCGTAAMATTGTCA |
|  | Primer-R | GCWGGTTTTGGTCCTGTGA |
|  | Probe | CAACTCAGGAAACARG |
| **Rotavirus A** | Primer-F | ACCATCTWCACRTRACCCTCTATGAG |
|  | Primer-R | GGTCACATAACGCCCCTATAGC |
|  | Probe | AGTTAAAAGCTAACACTGTCAAA |
| **Norovirus GII** | Primer-F | CARGARBCNATGTTYAGRTGGATGAG |
|  | Primer-R | TCGACGCCATCTTCATTCACA |
|  | Probe | GAGGGSGATCGCRATCT |
| **Adenovirus** | Primer-F | CACTTAATGCTGACACGGGC |
|  | Primer-R | ACTGGATAGAGCTAGCGGGC |
|  | Probe | TGCACCTCTTGGACTAGT |

**Table S2: Counter measures implanted by the Kenyan government to counter the spread of COVID-19 in Kilifi**

| **Years** | **Measures** |
| --- | --- |
| **2020** | Travel restriction from other countries with reported COVID cases |
|  | Ban of social gatherings including churches, weddings, and funerals |
|  | Restriction of movement into and out of Kilifi county |
|  | Closure of schools |
|  | Compulsory wearing of masks in public |
|  | Ban of local air travel |
|  | Restriction of restaurants opening hours and offer take-away services only |
|  | Night curfew (7 p.m. to 5 a.m. then 11 p.m. to 4 a.m.) |
|  | Suspension of political gatherings and meetings |
| **2021** | Night curfew |
|  | Compulsory wearing of masks in public |
|  | COVID-19 vaccination launched |
|  | Cessation of all movement by air, rail, and road in disease infected areas |
|  | Suspension of political gatherings and meetings |
| **2022** | Compulsory wearing of masks in public (up to March 2022) |
|  | Compulsory hand washing (up to March 2022) |
|  | Social distancing (up to March 2022) |

**Table S3:** Enteric virus coinfections observed in Kilifi, Kenya between 2019 and 2022

|  | **2019** | **2020** | **2021** | **2022** |
| --- | --- | --- | --- | --- |
| **Coinfection** |  |  |  |  |
| Norovirus GII & Adenovirus_40/41 | - | 1 | 1 | - |
| Norovirus GII & Sapovirus | - | 1 | - | - |
| Rotavirus A & Adenovirus 40/41 | - | - | - | 1 |
| Rotavirus A & Astrovirus | - | - | - | 2 |
| Rotavirus A & Norovirus GII | 1 | - | - | 1 |
| Rotavirus A & Sapovirus | 1 | - | 1 | 2 |
| Sapovirus & Astrovirus | 2 | - | - | - |
